# Gastrointestinal image stitching based on improved unsupervised algorithm

**DOI:** 10.1371/journal.pone.0310214

**Published:** 2024-09-18

**Authors:** Rui Yan, Yu Jiang, Chenhao Zhang, Rui Tang, Ran Liu, Jinghua Wu, Houcheng Su

**Affiliations:** 1 College of Information Engineering, Sichuan Agricultural University, Ya’an, China; 2 College of Information Engineering, University of Macau, Macao, China; University of Science and Technology of China, CHINA

## Abstract

Image stitching is a traditional but challenging computer vision task. The goal is to stitch together multiple images with overlapping areas into a single, natural-looking, high-resolution image without ghosts or seams. This article aims to increase the field of view of gastroenteroscopy and reduce the missed detection rate. To this end, an improved depth framework based on unsupervised panoramic image stitching of the gastrointestinal tract is proposed. In addition, preprocessing for aberration correction of monocular endoscope images is introduced, and a C2f module is added to the image reconstruction network to improve the network’s ability to extract features. A comprehensive real image data set, GASE-Dataset, is proposed to establish an evaluation benchmark and training learning framework for unsupervised deep gastrointestinal image splicing. Experimental results show that the MSE, RMSE, PSNR, SSIM and RMSE_SW indicators are improved, while the splicing time remains within an acceptable range. Compared with traditional image stitching methods, the performance of this method is enhanced. In addition, improvements are proposed to address the problems of lack of annotated data, insufficient generalization ability and insufficient comprehensive performance in image stitching schemes based on supervised learning. These improvements provide valuable aids in gastrointestinal examination.

## Introduction

Gastrointestinal cancers, encompassing malignancies of the esophagus, stomach, liver, pancreas, and colorectum, pose a significant global health burden. Early detection and timely intervention are crucial in the fight against gastrointestinal cancers, as many of these cancers are largely preventable if precancerous polyps are idenpngied and removed during asymptomatic stages [1].

While targeted gastrointestinal screening is vital for cancer control, the diagnostic accuracy of existing techniques remains limited. Flexible endoscopy, the preferred method for examining the gastrointestinal tract, has inherent technical constraints. Even skilled endoscopists may miss up to 6% of terminal adenomas and up to 30% of all adenomas using standard white light colonoscopy [2].

These statistics underscore the need for advanced screening and diagnostic methods in gastrointestinal cancer. Developing more sensitive techniques is crucial for earlier detection, effective intervention, and better outcomes for patients.

Recent advancements in medical diagnostics have introduced novel technologies addressing the limitations of current endoscopes in cancer screening. Standard gastrointestinal endoscopy using white light imaging often fails to reveal critical diagnostic information [3], prompting the exploration of alternative imaging modalities and techniques.

One promising approach involves the use of computed tomography (CT) or magnetic resonance imaging (MRI) scans to examine the gastrointestinal tract [4]. These advanced imaging techniques enable the visualization of polyps, diverticula, and other diseases through segmentation and surface. By providing detailed three-dimensional representations of the gastrointestinal anatomy, these imaging modalities can potentially aid in the early detection and characterization of abnormalities, thereby improving diagnostic accuracy and patient outcomes.

The integration of arpngicial intelligence (AI) in gastroenterology has grown significantly over the past decade. Computer-aided diagnosis enhances the detection and differentiation of colon polyps during colonoscopy [5]. Su et al. [6] proposed a multi-branch feature fusion network (MBFFNet) for real-time segmentation of colonoscopy images, outperforming the U-net architecture in terms of parameters, FLOPS, and Madds.

Traditional medical imaging techniques provide limited views, hindering complete organ observation. Image stitching can create a comprehensive view of the gastrointestinal tract, aiding in the accurate diagnosis of lesions. Despite the rise of deep learning, research on image stitching in medical imaging remains scarce. The gastrointestinal tract’s low-textured appearance, varying lighting conditions, and diverse backgrounds challenge traditional feature descriptors like SIFT [7] and HOG [8].

To address several of these issues, this study constructed an unsupervised gastrointestinal image stitching framework. For general endoscopic lens correction processing, Hough transform (HT)-based radial aberration correction is used to enhance aberrated image processing in different optical lenses. In addition, in order to improve the accuracy and feature extraction capability, we combined modules such as C2f in YOLOV8 [9]. Our model successfully accomplishes the stitching of gastrointestinal images, even in complex background scenes, while maintaining high accuracy. Evaluation of the dataset confirms the excellent performance of the proposed model over other models, achieving good results at different resolutions. The main contributions of this paper can be summarized as follows:

1. A coarse categorization of the gastric region into distinct categories is performed, and a splice is made for each region. This generates a controlled, clear, and colorful map of the mucosal regions of the stomach, allowing for a more intuitive observation of the results and avoiding missed detections.
2. An unsupervised image stitching method is applied to medical images to circumvent the need for labeling, thereby reducing the workload and cost associated with manual labeling.
3. Data preprocessing for endoscopic image aberration correction is incorporated to mitigate aberrations caused by lens factors, while the C2f module is introduced to enhance feature extraction capability, enabling the model to pay more attention to texture feature processing and improving the actual accuracy of stitching.
4. Ghosting arpngacts produced by image stitching may obscure or blur real anatomical structures or lesions. Therefore, the reconstruction stage from feature to image in the unsupervised image stitching model is employed to eliminate these arpngacts, which is crucial for accurate lesion assessment.

## Related work

### Feature-based image stitching

Image stitching algorithms have been extensively studied and developed to address the challenges of combining multiple images into a seamless panoramic view. In the context of gastrointestinal endoscopy, these algorithms play a crucial role in enhancing the visualization and analysis of the digestive tract.

Feature-based image stitching algorithms, such as SIFT [7], are known for their robustness against viewpoint changes, scale variations, and rotations but are computationally expensive for real-time endoscopic procedures. Improved algorithms, like Liu et al.’s [10] combination of nearest neighbor matching with RANSAC, enhance feature point matching accuracy but still struggle in feature-sparse regions common in gastrointestinal endoscopy. SURF [11] offers faster feature extraction and matching than SIFT, but at the cost of reduced matching points and lower tolerance to scaling and rotation changes, problematic in endoscopic applications with frequent viewpoint variations. Chen et al. [12] proposed a SIFT-based algorithm tailored for intestinal imaging, reducing dimensionality and computing principal directions, showing promising results in gastrointestinal endoscopy. APAP (As-Projective-As-Possible) [13] better handles perspective transformations and reduces deformation and distortion compared to traditional methods but requires iterative optimization and may exhibit arpngacts near boundaries with significant depth changes, challenging in complex endoscopic scenes.

Feature-based algorithms can achieve natural stitching results but depend heavily on feature quality, often failing in gastrointestinal scenes with few distinguishable features and lower resolution. This limitation underscores the need for algorithms tailored to endoscopic applications, addressing the unique challenges of gastrointestinal imaging.

### Learning-based image stitching

Image stitching, the process of combining multiple images into a larger panorama, faces challenges in data labeling and generalization of complex scene models. Accurately labeling real datasets for image stitching is difficult, complicating the use of deep learning methods. Despite their powerful feature extraction capabilities, these methods need further development to handle complex scenes effectively.

Nie et al. [14] introduced a valuable learning-based framework for arbitrary view image stitching. This framework encompasses three main components: homography estimation, spatial transformation, and content refinement. However, it is worth noting that this method is limited to handling low-resolution input and may not yield satisfactory results in practical applications. While an important step forward, further advancements are needed to enhance its overall performance. In addition to the aforementioned framework, Nie et al. [15] also proposed the concept of large baseline deep homography. This concept expands the scope of homography estimation, enabling the handling of larger viewpoint differences and perspective transformations. Furthermore, they introduced an edge-preserving stitching method that prioritizes the preservation of edge information during the stitching process. These contributions provide valuable insights into improving the robustness and visual quality of stitched images. Nie et al. [16] proposed a deep rectangle solution for image stitching, generating rectangular images in a residual progressive manner. This method addresses irregular boundaries but introduces projection distortion. Additionally, unlike unsupervised models, it requires a pre-constructed dataset with irregular boundaries and scenes, making data preparation cumbersome. Nie et al. [17] proposed a parallax-tolerant deformation model that avoids complex geometric features. The iterative strategy reduces arpngacts effectively but increases runtime, taking approximately 0.1 seconds per iteration. Jia et al. [18] proposed an unsupervised image stitching framework, where a coarse-to-fine unsupervised image stitching network can achieve pixel-level alignment, and a global transformation is generated by iterative dense feature matching combined with an error control strategy to mitigate the differences introduced by large parallax. However, in endoscopy, the parallax is usually not large, and the introduction of a large-scale feature extractor will bring unnecessary overhead.

Overall, while deep learning has shown promise in image stitching, significant work is required to overcome the difficulties associated with data labeling and generalization. The proposed methods discussed above contribute valuable insights, but practical applications still demand further development and improvement.

### Distortion recpngication

Radial distortion is a common lens distortion that causes straight lines in an image to become curved lines. In recent years, deep learning methods have made significant progress in correcting radial distortion.

Liao et al. [19] introduced the first end-to-end trainable adversarial radial distortion correction framework, known as DRGAN. This framework employs adversarial training from low to high perceptual loss, allowing it to learn the mapping between images of varying structures. Additionally, it supports unlabeled training and one-step correction. However, DRGAN’s reliance on a synthetic distorted image dataset from a specific camera model results in suboptimal performance when applied to images from other cameras. Liao et al. [20] proposes a general single-shot distortion correction algorithm framework, addressing the challenge of single-shot distortion correction by constructing a distortion distribution map (DDM) to unify different camera models into the same domain. This model-free approach enhances generalization. However, the resulting corrected image is irregular, complicating subsequent image stitching. Yang et al. [21] proposed a feature-level correction scheme within a distortion recpngication network, embedding a correction layer in the skip-connection and utilizing appearance flow for pre-correcting image features. Experimental results demonstrate the method’s superiority across various datasets. However, it produces non-rectangular corrected images, posing challenges for subsequent image stitching. Liao et al. [22] designed a local-global associated estimation network that learns ordinal distortion to approximate realistic distortion distribution, using image patches for efficient distortion recpngication. However, further customization and optimization are needed for specific distortions or scenarios.

Although deep learning has advanced radial distortion correction, several challenges remain: 1) Established Reliability: Traditional geometric calibration algorithms are well-understood and widely accepted, providing predictable results. 2) Implementation Complexity: Deep learning methods are complex and computationally intensive, requiring extensive resources. 3) Data Requirements: Large amounts of labeled training data are necessary, which is challenging for medical imaging. Given these factors, we prioritized a proven, straightforward approach for our gastrointestinal imaging study to ensure reliable application within our constraints.

## Materials

To train our network, we propose GASE-Dataset, a dataset for gastroenteroscopy image stitching. The construction of data sets mainly involves data set collection and data set generation.

### Data collection

**Fig 1:**
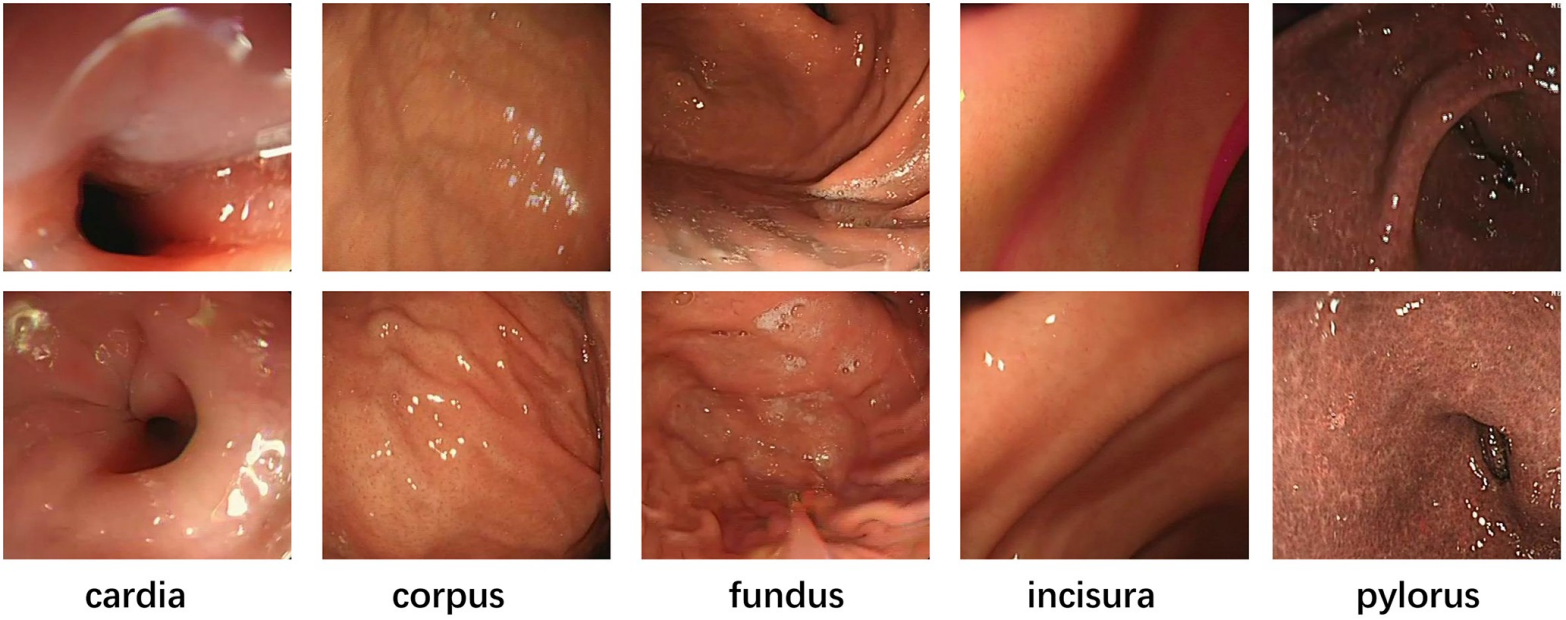
Biopsy pictures of different parts.

**Fig 2:**
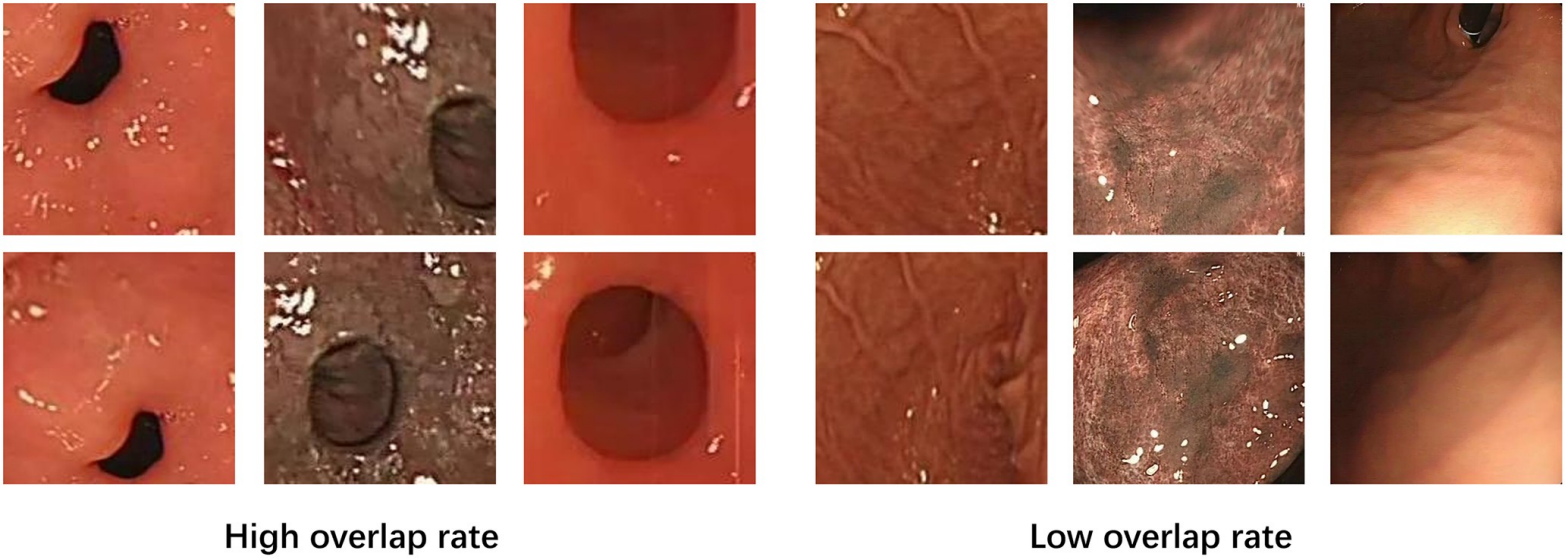
Different overlap rates of biopsy pictures.

**Fig 3:**
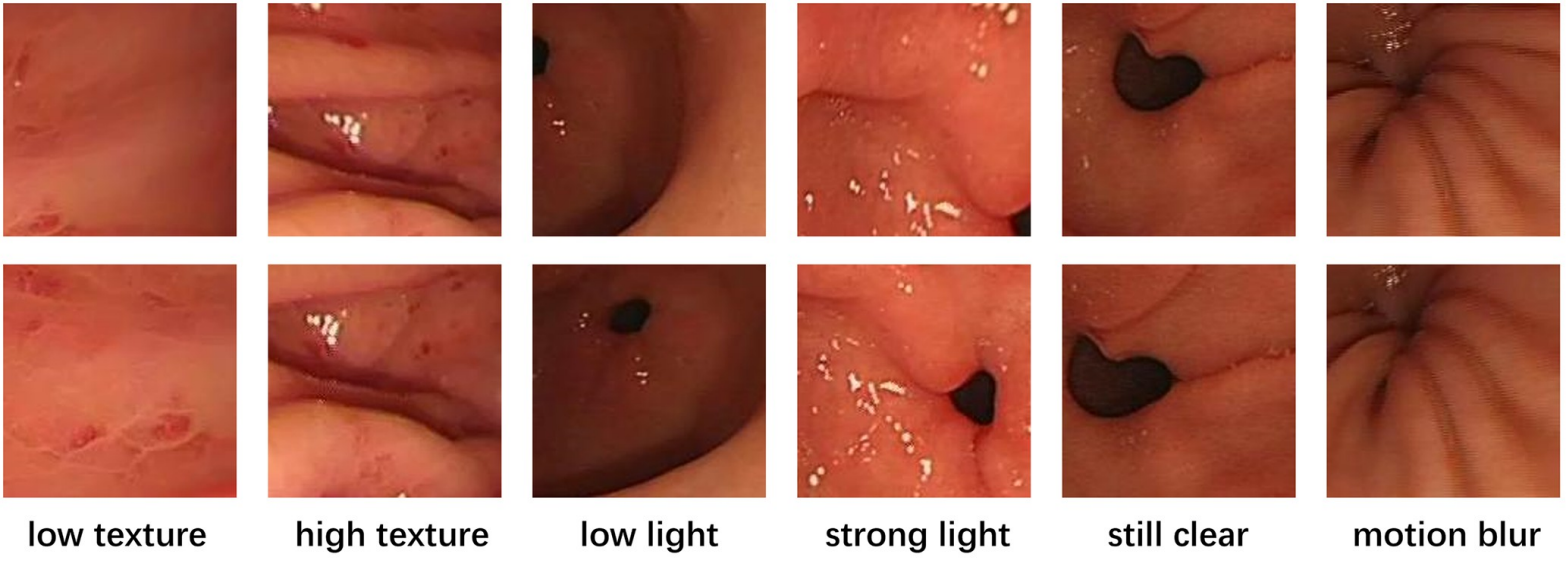
Biopsy images under different circumstances.

The raw data used in this study was obtained from endoscopic examinations conducted at the Gastroenterology Department of Kiang Wu Hospital in Macau. A recording license was obtained for the data, and variable-motion videos were captured using the EVIS EXERA III GIF-H190N gastrointestinal endoscope. The optical system of this endoscope has specific parameters, including a field of view of 140° and a depth of field ranging from 3 to 100 mm. To enhance the model’s robustness, we selected different sections for examination (Fig 1) and extracted frames from these videos at various intervals. It was crucial to collect samples with different overlap rates(We define high overlap as an image overlap greater than 60%, and low overlap as an image overlap less than 60%.) to ensure the dataset’s quality and applicability (Fig 2). Additionally, to comprehensively evaluate the complexity of the gastrointestinal tract, the dataset includes various scenarios commonly encountered during endoscopy, such as low texture, high texture, low light, strong light(Images with an average pixel intensity exceeding 200 are defined as bright light images.), clear images(Motion blur can be quanpngied by pixel shift, with noticeable blur occurring when the shift exceeds 1 pixel during exposure.), and motion blur(Fig 3).

In this study, a series of gastrointestinal examinations were conducted using an endoscope, and corresponding videos were recorded. The total duration of these videos amounts to 35 minutes and 4 seconds, excluding the start-up and shut-down periods of the device. Consequently, the effective duration of the captured footage was determined to be 30 minutes and 33 seconds. The recorded video files were then sampled and sliced to generate a raw image dataset, resulting in a total of 52,886 images. The dataset was divided into a training set, consisting of 47,597 cases, and a test set, consisting of 5,289 cases. In our dataset, different parts of the gastrointestinal tract, namely the cardia, corpus, fundus, incisura, parsPylorus_antrum, and pylorus, account for approximately 5%, 12%, 18%, 7%, 31%, and 26% respectively, arranged in descending order.

### Data generation

**Fig 4:**
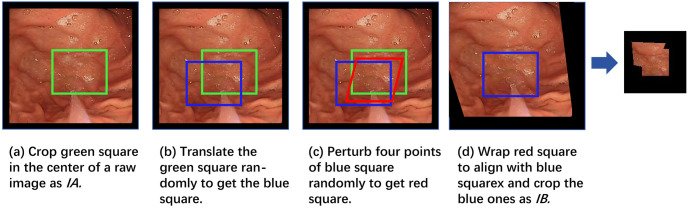
Deep stitching network dataset generation.

In order to simulate the image input of any view during gastrointestinal examination, we employ the synthetic data set method proposed by Nie et al. [14] to create a data set composed of four tuples (*I*_*A*_, *I*_*B*_, *f*, *label*) for image stitching. The specific process of data set generation is illustrated in Fig 4. This frees depth image stitching from the constraints of image views.

## Methods

In this section, we describe our methodologies for addressing the challenges of endoscopic distortion and gastrointestinal image stitching. To correct image distortion resulting from the optical characteristics of endoscopes, we employ a radial distortion correction technique based on the Hough Transform (HT). This method requires no prior calibration, allowing for its direct application during the acquisition or storage of video data. Additionally, it is camera-agnostic, making it versatile across various imaging devices. For image stitching, we introduce an enhanced approach that integrates the C2f module from Yolov8 with UNDIS to augment the feature extraction capabilities of the model. UNDIS operates in two phases: initial coarse alignment followed by detailed reconstruction. This two-stage process transforms feature data back into pixels, effectively mitigating ghosting arpngacts and yielding superior results.

### Distortion correction

The preprocessing step involving aberration correction is crucial for ensuring the accuracy and quality of the subsequent image stitching process. In our methodology, we employ a Hough transform-based radial distortion model to correct lens aberrations, particularly radial distortion, which is a common issue in endoscopic imaging.

#### Radial distortion correction based on Hough transform

To correct radial distortion, we utilize a Hough transform-based model. The Hough transform is a robust feature extraction technique that idenpngies geometric shapes, such as lines and circles, within an image. In the context of radial distortion correction, the Hough transform helps in accurately detecting and mapping the distorted features to their correct positions. Building upon the defined concept of radial distortion, the proposed method comprises two primary steps:

1. Automatically idenpngying candidate lines;
2. Iteratively adjusting distortion parameters to enhance the straightness of these lines.

To detect straight lines, we utilize the Hough Transform (HT) [23], a well-established image processing technique for idenpngying geometric shapes. This method is employed in both of the aforementioned steps. The initial phase involves locating the region of interest (ROI) where distortion is most apparent. To manage variations across different images, we adopt an automated iterative approach.

**Fig 5:**
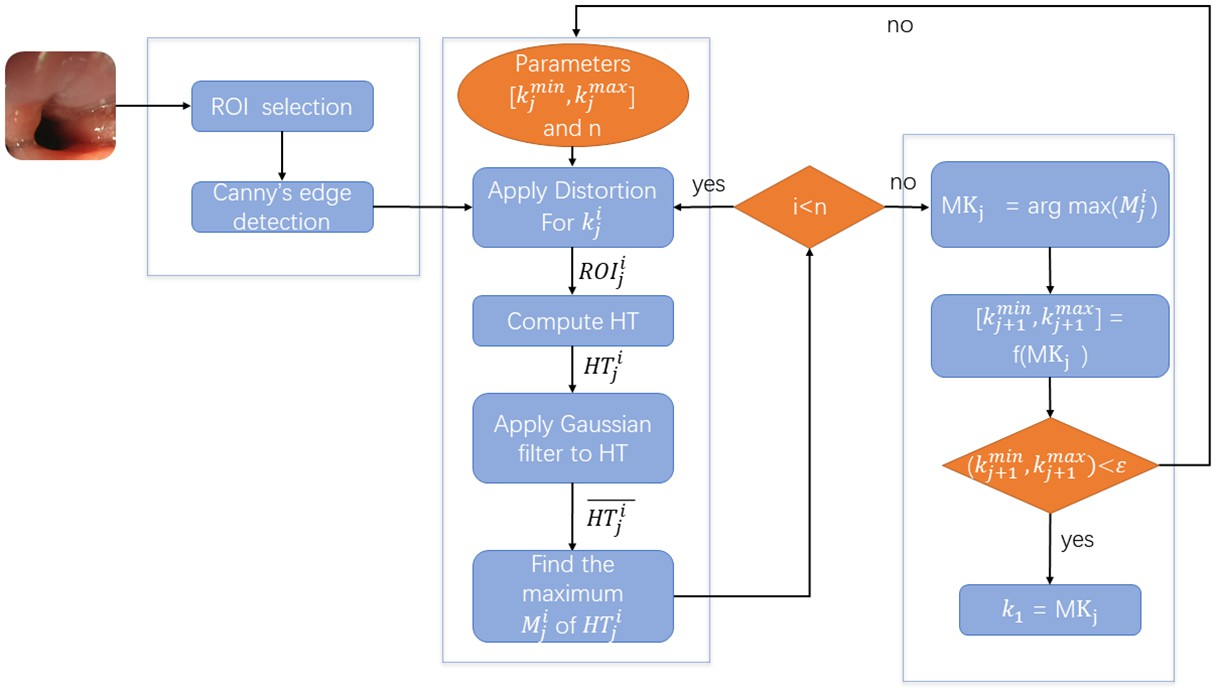
Flow chart of the proposed system.

In our study, the Canny edge detector [24] is employed on the extracted region of interest (ROI) to isolate “strong” edges via a non-maximum suppression technique. Subsequent to the edge detection, distortion is applied iteratively to the ROI using a multi-resolution strategy. As illustrated in Fig 5, the procedure begins with the initial bounds *k*^*min*^ and *k*^*max*^. The range between these bounds is divided into *n* steps. For each step *i*, the distortion parameters are determined as follows:

$$\begin{array}{c} k^{i}=k^{\text{min}}+i\frac{k^{\text{max}}-k^{\text{min}}}{n} \end{array}$$ (1)

By setting *k*_1_, we generate an “undistorted” version of the region of interest (*ROI*).

As illustrated in Fig 5, the subsequent step involves smoothing the Hough Transform (HT) space using a Gaussian filter to mitigate oscillations arising from quantization effects.

The system’s automated routine employs a method based on the Hough Transform to idenpngy regions characterized by high global straightness, which may be located distant from the center of distortion. The system only focuses the search on quasi-horizontal or quasi-vertical lines, that are $\theta \in [-\tau_{\theta },+\tau_{\theta }]\cup [\frac{\pi }{2}-\tau_{\theta },\frac{\pi }{2}+\tau_{\theta }]$. Correlate the obtained HT spaces to calculate the space $\overset{\cdot }{H}=\prod_{s}HT_{s}$. The parameter pair (*ρ*_0_, *θ*_0_) maximizing *H* an be used to apply an inverse transformation, thereby obtaining an approximate ROI in the image plane.

In summary, the aberration correction step using a Hough transform-based radial distortion model is pivotal for achieving high-quality image stitching in gastrointestinal endoscopy. By addressing and correcting radial distortion accurately, we can mitigate the risk of misalignments, blurring, and geometric inconsistencies, thereby enhancing the overall quality and diagnostic utility of the stitched images.

### Unsupervised coarse image alignment

Initially, we conduct a coarse alignment of the two input images. Specifically, for two gastrointestinal endoscopy images, we estimate the homography using an unsupervised deep learning network. Subsequently, these images are approximately aligned within the stitching-domain transformer layer.

#### Unsupervised homography constraints

**Fig 6:**
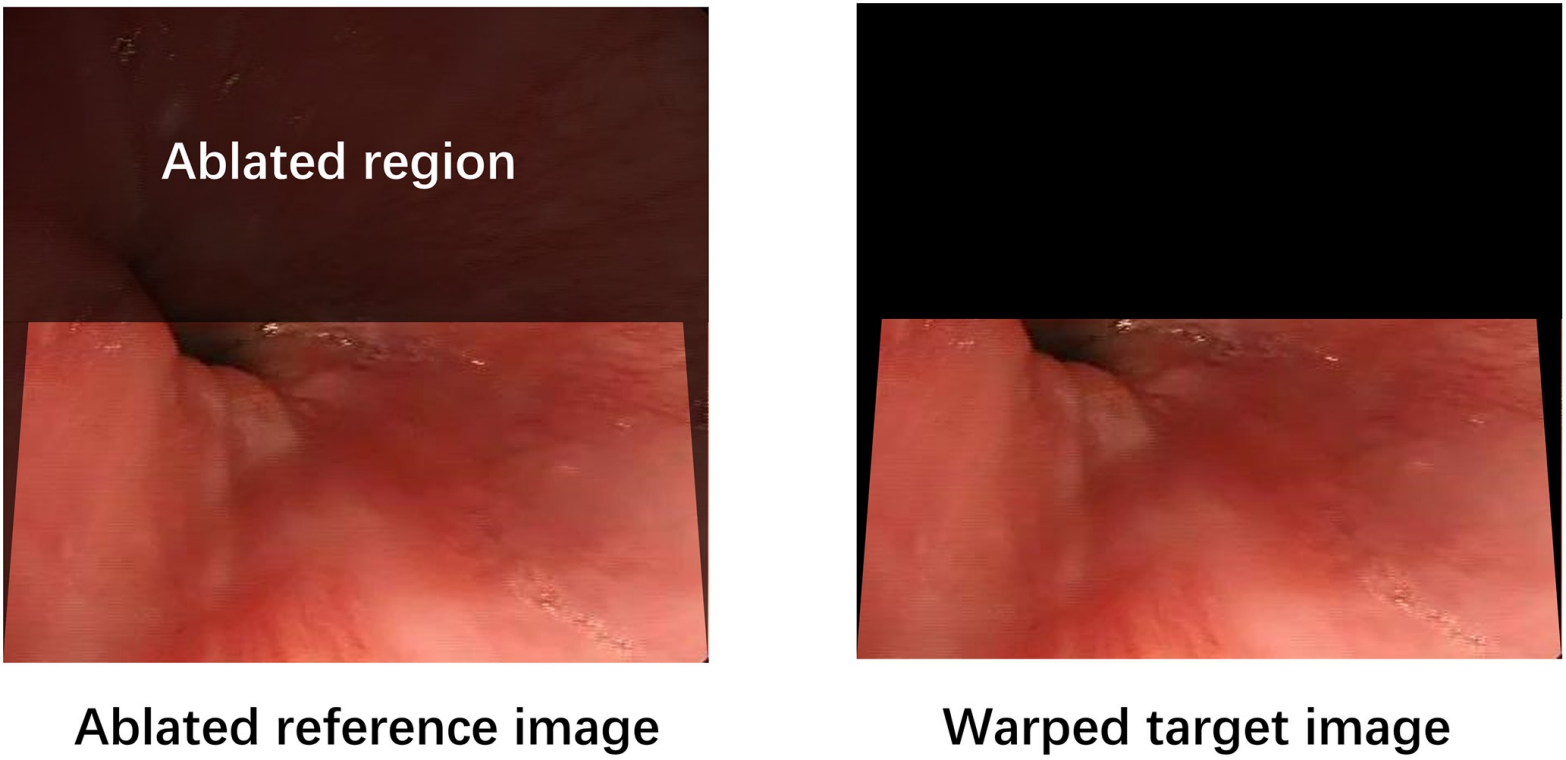
Ablation-based strategy.

We employ an ablation-based strategy [25] to enhance the precision of unsupervised homography estimation. Specifically, we use the entire image as input to ensure inclusion of all overlapping regions. The approach involves aligning the warped target image closely with the reference image and subsequently ablating regions in the reference image corresponding to invalid pixels in the warped target image, as illustrated in Fig 6. This method proves particularly effective in scenarios with large baselines, such as those encountered in gastroenteroscopy where disparities are significant, making ablation-based strategies particularly advantageous for unsupervised homography estimation.

The unsupervised homography objective function is expressed as Eq 2:

$$\begin{array}{cc} & L_{PW}^{\prime }={\left\|H\left(E\right)⊙I^{A}-H\left(I^{B}\right)\right\|}_{1} \end{array}$$ (2)

where ⊙ is the pixel-wise multiplication and *E* is an all-one matrix with identical size with *I*^*A*^.

#### Architecture of unsupervised homography network

To address a range of baseline scenarios, we employ the multi-scale deep model introduced by Nie et al. [15]. This model integrates feature pyramids and feature correlations within a unified framework, enabling the prediction of homography from coarse to fine scales.

#### Stitching domain transformer layer

**Fig 7:**
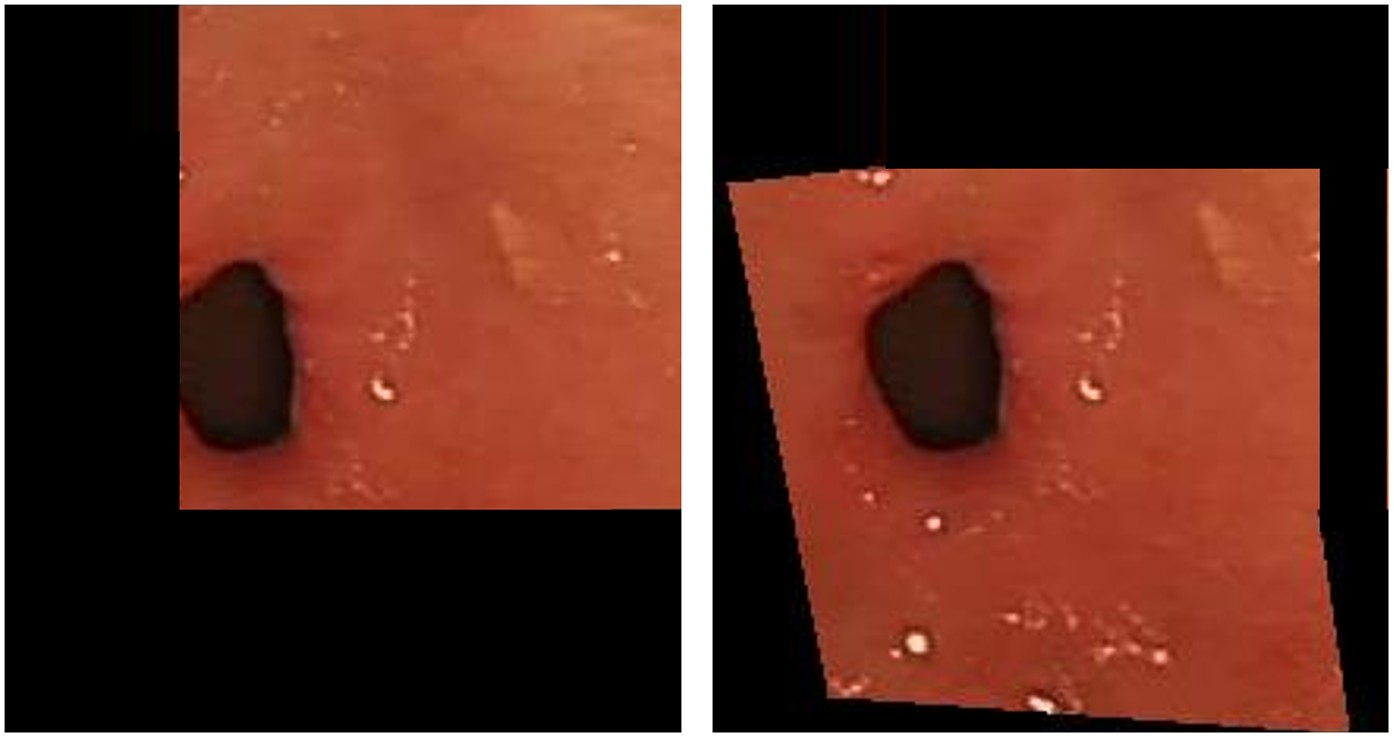
Stitching domain transformer layer distortion.

One of the challenges in stitching deep gastrointestinal tract images is the variation in overlap rates, which results in stitched images with different resolutions. To address this issue, we propose the use of a stitched domain transformation layer. The stitching domain is defined as the minimum bounding rectangle of the stitched image, which not only conserves space and time but also ensures the integrity of image content. The warping results are depicted in Fig 7, and the implementation of our seamed domain transformation layer is presented as follows.

First, we calculate the coordinates of the 4 vertices in the warped target image through Eq 3:

$$\begin{array}{c} \left(x_{k}^{W},y_{k}^{W}\right)=\left(x_{k}^{B},y_{k}^{B}\right)+\left(\Delta x_{k},\Delta y_{k}\right),k\in \left\{1,2,3,4\right\} \end{array}$$ (3)

The coordinates $\left(x_{k}^{W},y_{k}^{W}\right)$ and $\left(x_{k}^{B},y_{k}^{B}\right)$ represent the vertex coordinates of the *k*-th point in the deformed target image and the target image, respectively. The offset of the *k*-th vertex, denoted as (Δ*x*_*k*_, Δ*y*_*k*_), is estimated from the network.

Eq 4 can be used to determine the size of the warped image, denoted as (H* × W*).

$$\begin{array}{cc} & W^{*}=\underset{k\in \{1,2,3,4\}}{\text{max}}\left\{x_{k}^{W},x_{k}^{A}\right\}-\underset{k\in \{1,2,3,4\}}{\text{min}}\left\{x_{k}^{W},x_{k}^{A}\right\}\\ & H^{*}=\underset{k\in \{1,2,3,4\}}{\text{max}}\left\{y_{k}^{W},y_{k}^{A}\right\}-\underset{k\in \{1,2,3,4\}}{\text{min}}\left\{y_{k}^{W},y_{k}^{A}\right\} \end{array}$$ (4)

The vertex coordinates $\left(x_{k}^{A},y_{k}^{A}\right)$ represent the corresponding points in the reference image, with the same values as $\left(x_{k}^{B},y_{k}^{B}\right)$.

To complete the process, we assign specific pixel values from the input images (*I*^*A*^, *I*^*B*^) to the warped image pixels (*I*^*AW*^, *I*^*BW*^). This assignment can be expressed as shown in Eq 5.

$$\begin{array}{cc} & I^{AW}=W\left(I^{A},I\right)\\ & I^{BW}=W\left(I^{B},H\right) \end{array}$$ (5)

The identity matrix *I* and the estimated homography matrix *H* are utilized in the process. The transformation matrix W is a 3 × 3 matrix that distorts the image, with the stitching domain set to H* × W*.

By applying this transformation, the input image is effectively converted into the stitching domain space. This results in a reduction in the space occupied by the feature map in the subsequent reconstruction network. Additionally, this approach enables the stitching of higher resolution images, even when GPU memory is limited.

### Unsupervised image reconstruction with C2f module

**Fig 8:**
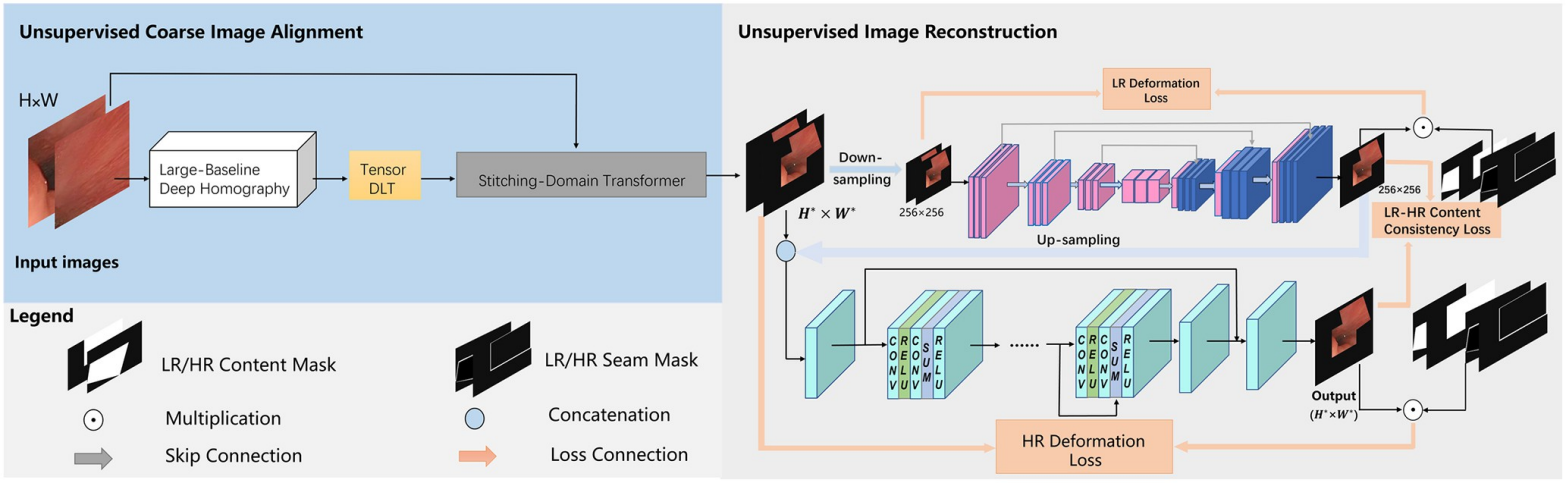
Image stitching framework.

A single homography is limited in its ability to represent the spatial transformation when dealing with input images of varying depths and viewing angles [26]. This limitation results in the occurrence of ghosting arpngacts during coarse image alignment, which is unacceptable in gastrointestinal image stitching. To mitigate the impact of ghosting, we incorporate a feature-to-pixel network with the C2f module to reconstruct the stitched image. The complete image stitching framework is illustrated in Fig 8.

#### Low-resolution deformation branch

To ensure that the network has a comprehensive understanding of misaligned regions, a low-resolution branch is employed initially to learn the deformation patterns for image stitching. On the right side of Fig 8, the warped image is downsampled to a resolution of 256 × 256. Subsequently, an encoder-decoder network with 3 pooling layers and 3 deconvolutional layers is utilized to reconstruct the stitched image. The convolutional layers have filter numbers of 64, 64, 128, 128, 256, 256, 512, 512, 256, 256, 128, 128, 64, 64, and 3 respectively. Additionally, skip connections are implemented to connect features with identical resolutions [27]. This effectively enhances the performance and convergence speed of deep networks.

**Fig 9:**
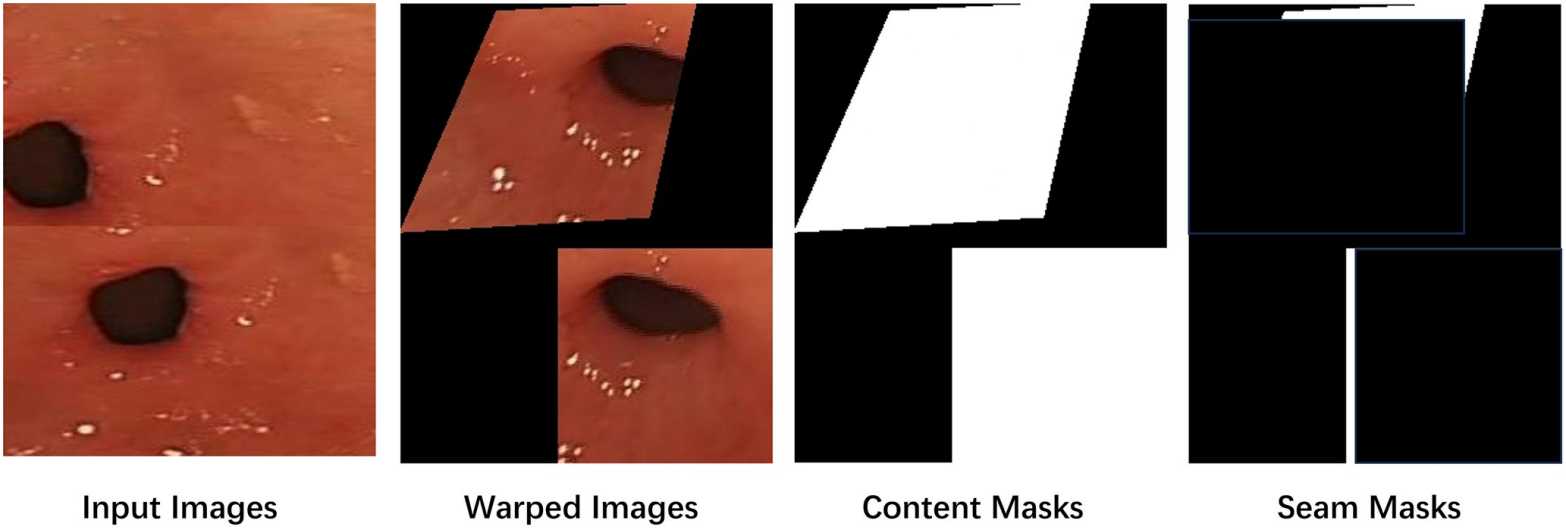
Learning deformation rules with masks in low resolution.

During this process, the deformation rules for image stitching are learned using two types of masks: content masks and seam masks (Fig 9). The content mask is employed to guide the reconstruction of the image, ensuring that it closely aligns with the features of the distorted image. On the other hand, the seam mask is utilized to preserve the natural and continuous appearance of the overlapping regions.

The content mask (*M*^*AC*^, *M*^*BC*^) is obtained using Eq 5, where we replace *I*^*A*^ and *I*^*B*^ with an all-1 matrix. The seam mask can be calculated using Eqs 6 and 7.

$$\begin{array}{c} \nabla M^{AC}=|M_{i,j}^{AC}-M_{i-1,j}^{AC}\left|+\right|M_{i,j}^{AC}-M_{i,j-1}^{AC}|\\ \nabla M^{BC}=|M_{i,j}^{BC}-M_{i-1,j}^{BC}\left|+\right|M_{i,j}^{BC}-M_{i,j-1}^{BC}| \end{array}$$ (6)

$$\begin{array}{c} M^{AS}=C\left(\nabla M^{BC}*E_{3\times 3}*E_{3\times 3}*E_{3\times 3}\right)⊙M^{AC}\\ M^{BS}=C\left(\nabla M^{AC}*E_{3\times 3}*E_{3\times 3}*E_{3\times 3}\right)⊙M^{BC} \end{array}$$ (7)

In the equations, (*i*, *j*) denotes the coordinate position, * represents the convolution operation, and C ensures that all elements are clipped between 0 and 1. The content loss and seam loss at low resolution can be expressed as Eqs 8 and 9 respectively.

$$\begin{array}{c} L_{Content}^{l}=L_{P}\left(S_{LR}⊙M^{AC},I^{AW}\right)+L_{P}\left(S_{LR}⊙M^{BC},I^{BW}\right), \end{array}$$ (8)

$$\begin{array}{c} L_{Seam}^{l}=L_{1}\left(S_{LR}⊙M^{AS},I^{AW}⊙M^{AS}\right)+L_{1}\left(S_{LR}⊙M^{BS},I^{BW}⊙M^{BS}\right) \end{array}$$ (9)

Here, *S*_*LR*_ represents the low-resolution stitched image. $L_{1}$ and $L_{P}$ correspond to the $L_{1}$ loss and perceptual loss respectively [28].

The total loss function for low-resolution unsupervised deformation is given by Eq 10.

$$\begin{array}{c} L_{LR}=\lambda_{c}L_{Content}^{l}+\lambda_{s}L_{Seam}^{l} \end{array}$$ (10)

λ_*s*_ and λ_*c*_ are content constraints and seam weighting coefficients respectively.

#### High-resolution refined branch

**Fig 10:**
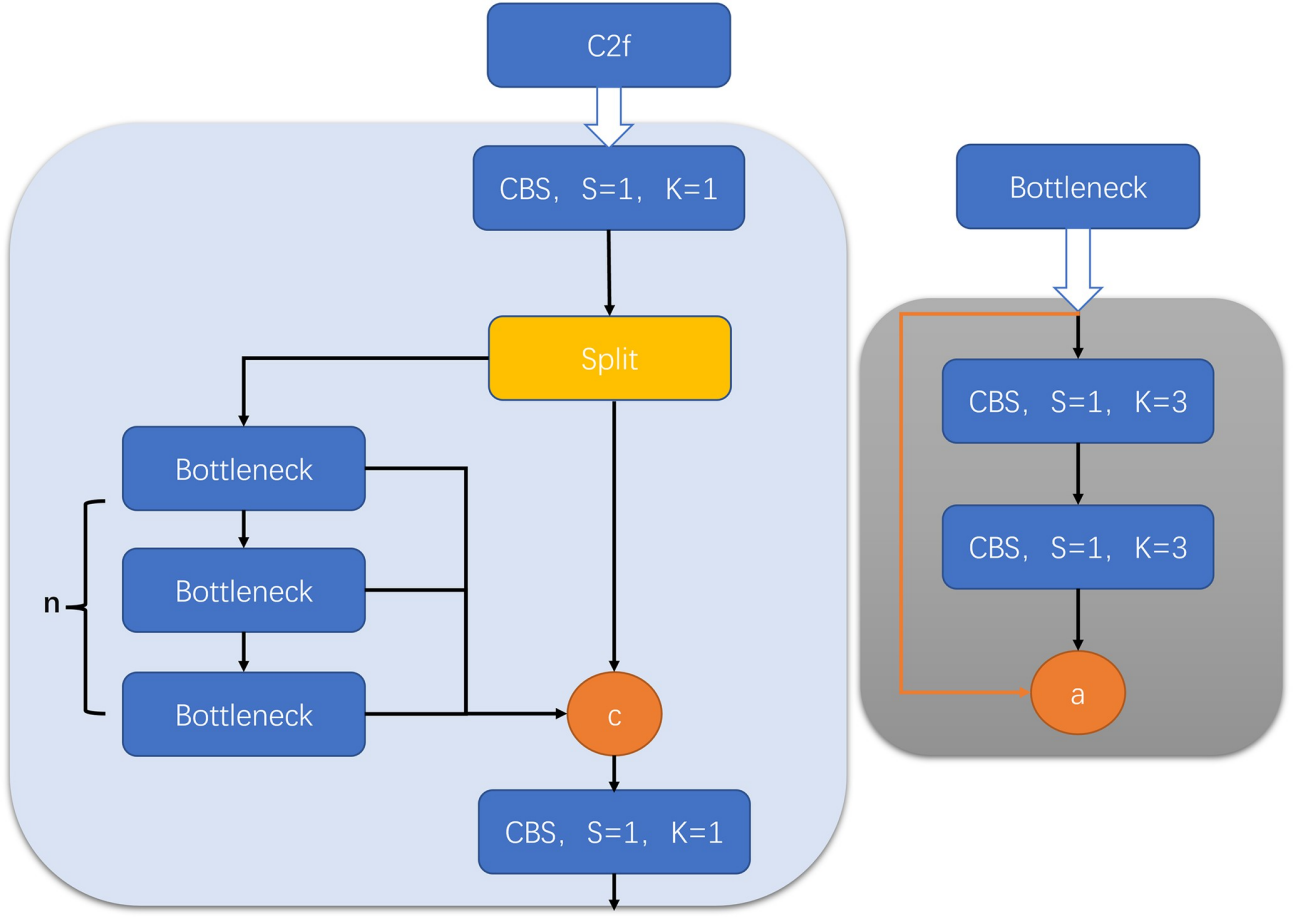
C2f structure diagram.

The high-resolution branch is utilized to refine the stitched image and address ghosting arpngacts. This branch consists entirely of convolutional layers, as depicted in the bottom part of Fig 8, and it is capable of handling images of any size. Specifically, it comprises three independent convolutional layers and eight resblocks [29]. Additionally, we introduce the C2f module, which is inspired by the C3 module [30] and incorporates ideas from ELAN [31]. The C2f module, illustrated in Fig 10 [9], effectively captures important image features, enhancing the accuracy and performance of the model.

In this branch, we upsample the *S*_*LR*_ to match the resolution of the warped image, and then concatenate them together as input. The output of this branch is the high-resolution refined stitched image *S*_*HR*_. The loss function for the high-resolution refined branch is given by Eq 11.

$$\begin{array}{c} L_{HR}=\lambda_{c}L_{Content}^{h}+\lambda_{s}L_{Seam}^{h} \end{array}$$ (11)

where $L_{Content}^{h}$ and $L_{Seam}^{h}$ represent the content loss and seam loss at high resolution, respectively. These losses are computed similarly to Eqs 8 and 9, with the difference being that the calculations are performed using *S*_*HR*_ and high-resolution masks instead of *S*_*LR*_ and low-resolution masks.

#### Objective function

To enable our network to simultaneously enhance resolution and eliminate parallax arpngacts, the content consistency loss is as in Eq 12:

$$\begin{array}{c} L_{CS}={\left\|S_{HR}^{256\times 256}-S_{LR}\right\|}_{1} \end{array}$$ (12)

where $S_{HR}^{256\times 256}$ is the resolution output in the low-resolution branch.

Considering all the relevant constraints, we can summarize the objective function for the image reconstruction stage as shown in Eq 13.

$$\begin{array}{c} L_{R}=\omega_{LR}L_{LR}+\omega_{HR}L_{HR}+\omega_{CS}L_{CS} \end{array}$$ (13)

Among them, *ω*_*LR*_, *ω*_*HR*_, and *ω*_*CS*_ represent the weight of each part.

### Experimental esnvironment and parameter adjustment

The training process for our unsupervised image stitching framework involves three steps. Firstly, we train a deep homography network on Stitched MS-COCO dataset [14] for 150 epochs. Secondly, we fine-tune the homography network on our proposed gastrointestinal tract image stitching dataset for 50 epochs. Lastly, we train a deep image reconstruction network on the gastrointestinal tract dataset for 20 epochs. We utilize the Adam optimization algorithm [32] with an initial learning rate of 10^−4^. The hyperparameters λ_*s*_ and λ_*c*_ are set to 2 and 10^−6^ respectively. The weights *ω*_*LR*_, *ω*_*HR*_, and *ω*_*CS*_ are set to 100, 1, and 1 respectively. For our experiments, the specific details of the experimental environment are provided in Table 1.

**Table 1:** Experimental environment configuration.

Category	Configuration
CPU	Intel(R) Core(TM) i5-12490F @3.00 GHz
GPU	GeForce RTX 4060
System enviroment	Windows11
Framework	Pytorch 1.13.1
CUDA-Toolkit	11.0
Programming version	Python 3.7

## Results

In order to assess the effectiveness of our algorithm, we performed a series of extensive experiments using the gastrointestinal stitching dataset. The evaluation process involved conducting a thorough analysis and comparison of our enhanced model with various well-known stitching models. Our evaluation focused specifically on evaluating the robustness, stitching quality, and speed metrics of the different models.

### Endoscopic correction experiment

The acquired gastrointestinal image data is prone to distortion due to the optical characteristics of the endoscopic lens. To address this, we need to perform image correction as a pre-processing step in gastrointestinal image stitching. To ensure the effectiveness of the correction process, we conducted experiments with different parameter settings using our dataset and selected the optimal parameters based on the results.

Specifically, we tested different values for *CAL*_*START*_*COEFF*, *CAL*_*COEFF*_*INC*_*FACTOR*, and *CAL*_*EPOCH*_*NUMB*. We tried values of 1e-9 and 1e-7 for *CAL*_*START*_*COEFF*, 9e-8 and 5e-8 for *CAL*_*COEFF*_*INC*_*FACTO*R, and uniformly set *CAL*_*EPOCH*_*NUMB* to 15. By comparing the experimental results, we determined the optimal parameter values and their corresponding *k* values. The results are summarized in Table 2.

**Table 2:** Effectiveness of different k values on quality.

	mode crop	A	B	C	D
Input resolution	CAL_START_COEFF	1.00E-09	1.00E-09	1.00E-07	1.00E-07
	CAL_COEFF_INC_FACTOR	9.00E-08	5.00E-08	9.00E-07	5.00E-07
	CAL_EPOCH_NUMB	15	15	15	15
	k	1.00E-09	2.71E-07	1.00E-07	6.00E-07
128x128	Average MSE	**5742.45**	5764.07	5813.57	5799.72
	Average RMSE	73.31	**73.23**	73.85	73.50
	Average PSNR	11.18	**11.22**	11.11	11.17
	Average SSIM	0.20	**0.20**	0.20	0.20
	Average RMSE_SW	55.79	**55.68**	56.37	56.12
256x256	Average MSE	5727.12	**5768.45**	5806.92	5803.83
	Average RMSE	**73.21**	73.26	73.80	73.53
	Average PSNR	11.19	**11.22**	11.11	11.16
	Average SSIM	**0.27**	0.27	0.27	0.26
	Average RMSE_SW	**53.79**	53.81	54.38	54.19
512x512	Average MSE	**5727.06**	5770.13	5806.45	5805.57
	Average RMSE	**73.21**	73.27	73.80	73.54
	Average PSNR	11.19	**11.22**	11.11	11.16
	Average SSIM	0.32	**0.32**	0.32	0.32
	Average RMSE_SW	**52.61**	52.63	53.17	52.96

Based on the results presented in Table 2, it is evident that, on average, using *CAL*_*START*_*COEFF* as 1e-9 and *CAL*_*COEFF*_*INC*_*FACTOR* as 5e-8 yields better performance compared to the other parameter combinations. Therefore, for subsequent experiments, we utilized the parameters from group B.

### Comparison of image stitching

To validate the effectiveness of our gastrointestinal image stitching method, we conducted a comparison with both traditional feature-based solutions and learning-based solutions. In this section, we selected LCP [33], APAP [13], and ELA [34] as representative feature-based solutions for the comparison with our algorithm.

#### Study on robustness

In order to evaluate the robustness of the methods in different scenarios, we conducted tests on a test set consisting of gastrointestinal tract image data collected using different endoscopes with varying resolutions due to the optical characteristics of the lens.

To simulate changes in the number of features, we resized the test set to different resolutions, namely 512 × 512, 256 × 256, and 128 × 128. As the resolution decreases, the number of features decreases accordingly.

Furthermore, current evaluations may not fully capture the variations and complexities encountered in different medical imaging scenarios. To further validate the capabilities of our model under diverse and more realistic conditions, we conducted supplementary experiments using the HyperKvasir dataset [35], a large and diverse collection of gastrointestinal images. HyperKvasir includes a wide range of endoscopic images with varying lighting conditions, anatomical structures, and pathological findings, making it an ideal testbed for further validating our method.

We selected a representative subset of the HyperKvasir dataset(3452 in total images), ensuring a mix of images with different characteristics such as lighting variations, tissue types, and motion arpngacts. Similar to our primary dataset, we applied the Hough transform-based radial distortion correction to the HyperKvasir images. The corrected images were then processed using our image stitching algorithm.

The results of the evaluations are presented in Table 3, where the “error” column indicates the number of program crashes and the “failure” column indicates the number of stitching failures.

**Table 3:** Robustness comparison of image stitching.

Input resolution	Metrics	APAP	ELA\mosaic_global	ELA\mosaic_rew	LCP	View-free	ours	HyperKvasir
128x128	Error	3188	4504	4557	5289	0	1	0
	Failure	35	52	48	0	0	0	0
	Geometric failure	82	125	84	0	0	0	48
	total	3305	4681	4689	5289	0	1	48
	Success rate	37.51	11.50	11.34	0	100	99.98	98.6
256x256	Error	2847	3111	3185	4617	/	0	0
	Failure	30	44	21	110	/	0	0
	Geometric failure	74	98	65	125	/	0	13
	total	2951	3253	3271	4852	/	0	13
	Success rate	44.2	38.49	38.15	8.26	/	100	99.6
512x512	Error	1699	3048	3036	4292	/	0	0
	Failure	25	25	51	44	/	0	0
	Geometric failure	65	83	98	56	/	0	11
	total	1789	3156	3185	4392	/	0	11
	Success rate	66.18	40.33	39.78	16.96	/	100	99.7

**Fig 11:**
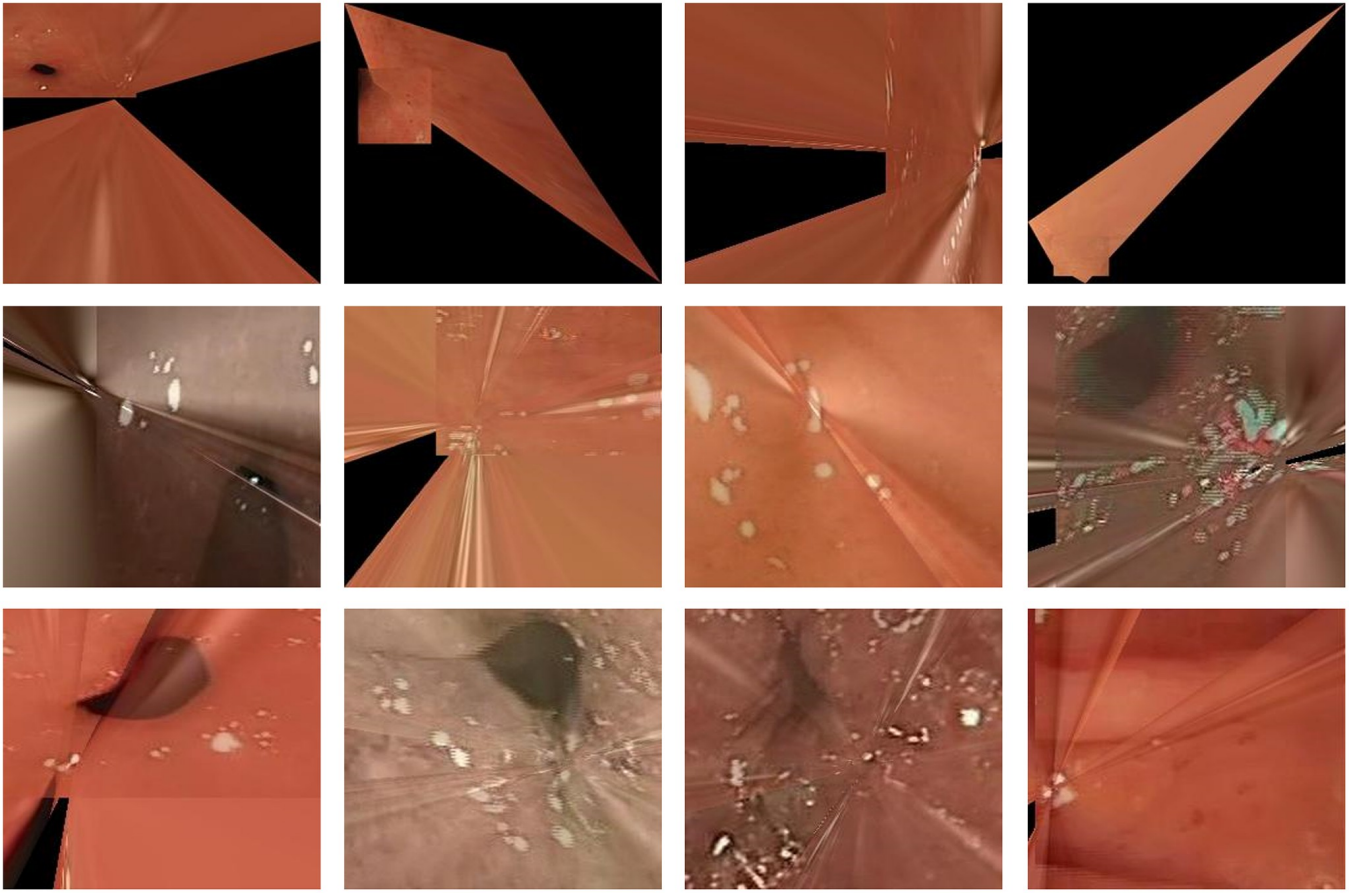
“Failed” examples. Top: failed geometric stitching; Second layer: obvious distortion; Bottom: intolerable ghosting.

In Fig 11, we provide visual examples of different types of stitching failures, including geometric stitching failure (top layer), significant distortion (second layer), and intolerable ghosting (bottom layer).

Upon examining the results, the following observations can be made:

1. Our method exhibits greater robustness compared to feature-based solutions. Notably, feature-based methods are susceptible to failures in gastrointestinal scenes with low lighting conditions and low texture, while our solution performs exceptionally well in such challenging scenarios, with minimal stitching failures.
2. As the resolution decreases, the success rate of learning-based methods decreases. However, our method demonstrates sustained robustness even with decreased resolution.
3. The initial evaluations demonstrated the efficacy of our method, the additional experiments on the HyperKvasir dataset provide further evidence of its robustness and applicability in real-world scenarios. In addition, the image quality of the model on the HyperKvasir dataset are shown in Table 4 and Fig 13 (below).

Feature-based solutions are highly sensitive to the number and distribution of feature points, which ultimately affects their performance and robustness across different scenarios. In contrast, our method successfully mitigates this issue thanks to the exceptional feature extraction capabilities of convolutional neural networks (CNN).

#### Study on visual quality

To verify the superiority of our image stitching method, we conducted a comparison with several representative algorithms in the field. Specifically, we compared our method with APAP, LCP, and ELA, as well as with feature-based solutions (referred to as view-free). However, it should be noted that the comparison with the view-free algorithm is unfair as our dataset is generated using a different method. Thus, we included it in the comparison for display purposes only.

**Fig 12:**
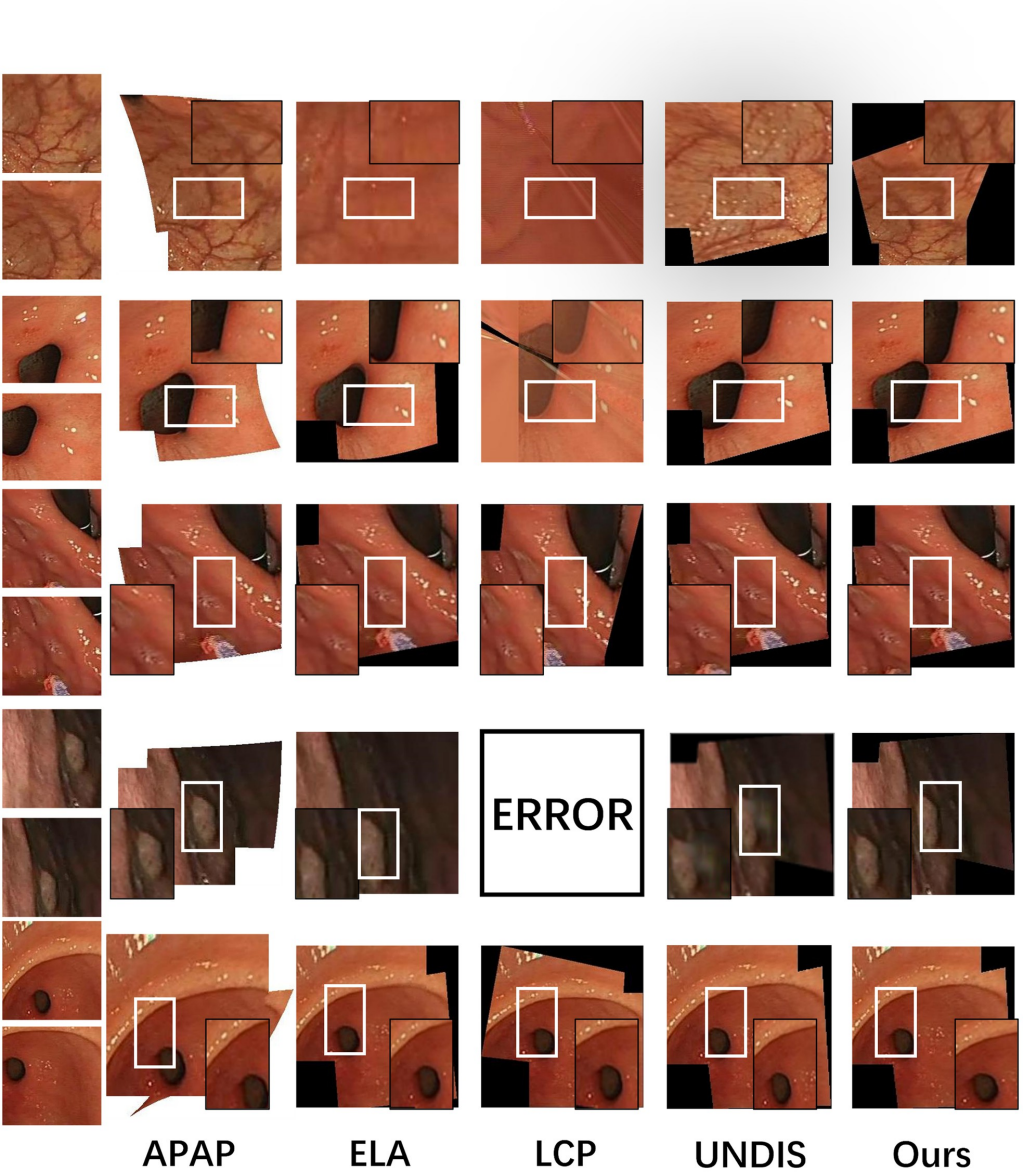
Comparison of image stitching quality.

In Table 4, the columns ‘resize’ and ‘crop’ indicate the normalization modes used for resizing and cropping respectively. The results show that our algorithm outperforms other tested image stitching algorithms in terms of MSE, RMSE, PSNR, SSIM, and RMSE_SW across various resolutions. The image stitching effects are demonstrated in Fig 12, providing specific details.

Our evaluation shows that the proposed algorithm consistently outperforms other tested image stitching algorithms across multiple metrics and resolutions. This is shown in Table 4. Our algorithm shows lower MSE and RMSE values, indicating a higher accuracy in image stitching. Higher PSNR values indicate better preservation of image quality. Superior SSIM scores reflect the structural similarity between the original and stitched images. The RMSE SW metric further confirms the robustness of our approach across various resolutions.

**Table 4:** Visual quality comparison of image stitching.

Input resolution	Metrics	APAP	ELA\mosaic_global	ELA\mosaic_rew	LCP	ours_crop	ours_resize	View-free	HyperKvasir
128x128	Average MSE	7712.44	7843.56	7829.87	/	5764.07	5639.74	258.28	10201.40
	Average RMSE	84.91	86.01	85.95	/	73.23	72.44	14.30	99.13
	Average PSNR	9.91	9.73	9.73	/	11.22	11.32	26.21	8.39
	Average SSIM	0.14	0.12	0.12	/	0.20	0.21	0.92	0.15
	Average RMSE_SW	66.90	70.51	70.44	/	55.68	54.94	4.02	80.57
256x256	Average MSE	7493.72	8004.84	7958.39	22046.85	5768.45	5659.61	/	10146.12
	Average RMSE	83.63	86.64	86.53	148.48	73.26	72.56	/	98.85
	Average PSNR	10.04	9.69	9.69	4.70	11.22	11.30	/	8.41
	Average SSIM	0.19	0.17	0.17	0.18	0.27	0.28	/	0.22
	Average RMSE_SW	65.36	69.26	69.12	114.37	53.81	53.23	/	77.24
512x512	Average MSE	7463.97	8210.93	8189.81	15113.15	5770.13	5677.43	/	10142.16
	Average RMSE	83.47	87.73	87.71	122.94	73.27	72.68	/	98.83
	Average PSNR	10.06	9.58	9.58	6.34	11.22	11.29	/	8.42
	Average SSIM	0.23	0.21	0.21	0.19	0.32	0.33	/	0.28
	Average RMSE_SW	64.92	69.76	69.69	85.44	52.63	52.15	/	75.55

#### Study on time cost

The speed of gastrointestinal image stitching plays a crucial role in clinical practice as it directly impacts the efficiency of doctors and the accuracy of disease assessment.

We conducted tests using images of resolutions 128 × 128, 256 × 256, and 512 × 512. As shown in Table 5, the time consumption of each algorithm increases as the image resolution increases.

**Table 5:** Time cost comparison of image stitching.

Input resolution	APAP	ELA\mosaic_global	ELA\mosaic_rew	LCP	View-free	ours_crop	ours_resize	Ours with Correction
128x128	1.6530s	0.0725s	0.0842s	/	0.4492s	0.0277s	0.0285s	0.0718s
256x256	3.1673s	0.1086s	0.1120s	0.5021s	/	0.0326s	0.0321s	0.0754s
512x512	14.4687s	0.4438s	0.4709s	2.5291s	/	0.0526s	0.0451s	0.0885s

Traditional image stitching algorithms typically involve optimizing and adjusting the matched feature points, which require operations such as image coordinate transformation and pixel adjustment in overlapping areas. These operations demand substantial computational resources and time. Particularly for our gastrointestinal tract dataset, traditional algorithms often encounter scenarios with multiple views or complex scenes, leading to a higher number of feature points and matching situations. This increases the computational effort and complexity of the algorithm, ultimately resulting in slower speeds.

Although the time cost of our method may not meet the stringent requirements of real-time video processing. But real-time can vary depending on the application. For gastrointestinal endoscopy detection, the application may prioritize accuracy over speed, making the processing time of each frame slightly higher. In addition, the listed methods have optimization potential. With further improvement of hardware acceleration, the processing time of each frame can be reduced to meet better requirements.

#### Ablation experiment

To validate the effectiveness of the improved algorithm, this study incorporates the C2f module as a feature extraction enhancement mechanism and introduces endoscopic correction as a data preprocessing step. These enhancements are integrated into the unsupervised image stitching algorithm, and experiments are conducted using our dataset. The experimental results are summarized in Table 6.

**Table 6:** Effectiveness of modules(Ablation experiment results).

Input resolution	Metrics	baseline_crop	baseline_resize	baseline+C2f_crop	baseline+C2f_resize	baseline+C2f+Correction _crop	baseline+C2f+Correction _resize
6*128x128	Average MSE	5931.198	5817.317	5817.053	5816.061	5816.974	5814.418
	Average RMSE	74.465	73.695	74.379	73.719	72.902	73.744
	Average PSNR	11.048	11.039	11.057	11.138	11.124	11.993
	Average SSIM	0.204	0.207	0.205	0.207	0.208	0.218
	Average RMSE_SW	56.769	55.993	55.683	55.970	55.062	55.655
	time_cost	0.034	0.028	0.036	0.036	0.053	0.054
6*256x256	Average MSE	5867.296	5867.296	5826.568	5826.969	5817.831	5813.538
	Average RMSE	74.071	74.071	73.449	73.608	72.902	72.938
	Average PSNR	11.092	11.092	11.048	11.880	11.125	11.121
	Average SSIM	0.269	0.269	0.278	0.270	0.284	0.345
	Average RMSE_SW	54.275	54.275	53.588	53.444	53.110	52.857
	time_cost	0.033	0.032	0.042	0.040	0.059	0.059
6*512x512	Average MSE	5861.613	5824.252	5850.726	5831.028	5822.269	5831.840
	Average RMSE	74.034	73.793	73.605	73.410	72.734	72.794
	Average PSNR	11.097	11.126	11.029	11.053	11.146	11.139
	Average SSIM	0.338	0.330	0.340	0.339	0.343	0.346
	Average RMSE_SW	53.875	53.642	53.331	53.127	52.766	52.724
	time_cost	0.049	0.049	0.053	0.054	0.076	0.083

In evaluating the graphics splicing effect, metrics such as MSE, RMSE, PSNR, SSIM, and RMSE_SW are utilized. Additionally, the time consumption of the algorithm is compared before and after the inclusion of the C2f module.

Compared with the original algorithm, after the introduction of the C2f module and endoscopic correction mechanism, when the resolution is 128x128, MSE dropped by 1.93%, RMSE dropped by 0.97%, RMSE_SW dropped by 3%, PSNR increased by 8%, and SSIM improved 6.8% (the lower the MSE, RMSE and RMSE_SW values, the better, the higher the PSNR and SSIM values, the better) When the resolution is 256 × 256 and 512 × 512, our improved models have improved to varying degrees, and the time consumption is within 0.1s, which meets the real-time requirements.

Based on the comparison and analysis of the experimental results, it can be concluded that the algorithm proposed in this paper outperforms the original algorithm. The incorporation of the C2f module not only preserves the lightweight nature of the model but also enhances gradient flow information, leading to better feature capture, improved accuracy, and enhanced overall performance.

**Fig 13:**
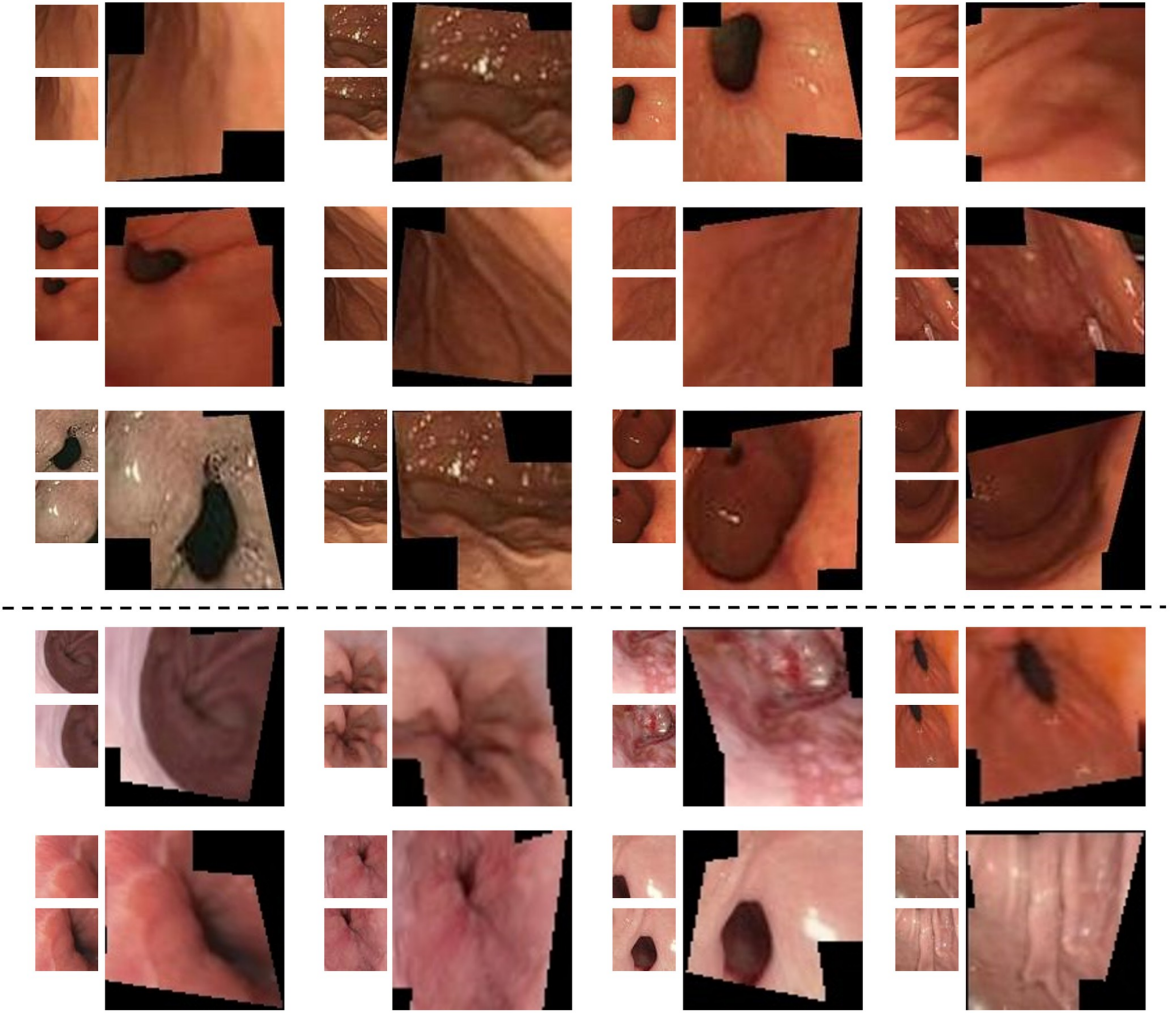
Image stitching results.

In Fig 13, we present the stitching effects achieved by the improved unsupervised stitching algorithm on the gastrointestinal tract dataset.

## Discussion

In the context of gastrointestinal image stitching, several challenges need to be addressed. First, endoscopy is a three-dimensional process that differs from the two-dimensional approach commonly employed in other imaging techniques. This makes it challenging to create a comprehensive map. Second, conventional images are typically captured at a viewing angle of 0-30 degrees, which minimizes the impact of viewing angle deviation, image distortion, and the difficulty of stitching. In contrast, endoscopes are subject to significant viewing angle deviation, image distortion, and greater difficulty in stitching together a complete image due to the optical factors of the lens and the short distance from the stomach. Lastly, peristaltic motion, low texture appearance, and varying intensity of lighting conditions in the gastrointestinal tract may lead to quality problems such as image blurring, arpngacts, and motion blur. This makes it difficult to extract features and prone to failures when stitching images.

In summary, this study aims to explore the optimal algorithm for real-time gastrointestinal image stitching under various conditions, and deploy the network model to hardware devices for practical application in medical detection.

## Limits and future work

It is important to acknowledge the limitations of this study. Firstly, the study did not take into account potential interference caused by personnel operations during the image acquisition process. For instance, variations in the examining physician’s detection techniques and conditions may affect the quality and representativeness of the data used for image stitching. In future research, it would be beneficial to collect a more diverse and representative gastrointestinal dataset under various conditions.

Secondly, the proposed solution is primarily based on homography-based stitching methods. This approach may encounter challenges when dealing with data that exhibits significant parallax, leading to reduced alignment performance. To address this, future work could focus on enhancing the alignment performance of the network, thereby reducing the burden on the image reconstruction process.

## Conclusion

In endoscopic images, the presence of many similar areas with low texture can pose challenges to achieving satisfactory stitching results. To address this issue, we propose an improved unsupervised stitching algorithm.

First, we apply the Hough Transform algorithm to correct the endoscopic image. This correction step helps align the images and improve the subsequent stitching process. Next, we estimate the homography using an unsupervised deep network. Coarse image alignment is achieved through the stitching-domain transformer layer, which helps handle the variations in the endoscopic images. This step is crucial to alleviate ghosting arpngacts that can occur during coarse alignment, these arpngacts are particularly undesirable in gastrointestinal image stitching. To overcome limitations associated with using homography alone, we introduce the C2f feature extraction enhancement module. This module enables reconstruction of the spliced image from features to pixels, and the progressive fade-out fusion technique is employed to achieve seamless stitching of endoscopic images.

Experimental results demonstrate that our improved unsupervised stitching algorithm produces better image quality compared to traditional algorithms. Additionally, the stitching time remains within an acceptable range, satisfying the real-time requirements for endoscopic image stitching. Multiple experiments were conducted to further verify the performance of the enhanced algorithm, and the results confirm its effectiveness in improving the accuracy of image stitching.
